# Graphene platelets enhanced pressureless- sintered B_4_C ceramics

**DOI:** 10.1098/rsos.171837

**Published:** 2018-04-11

**Authors:** Dezhi Gao, Jie Jing, Jincheng Yu, Xue Guo, Yubai Zhang, Hongyu Gong, Yujun Zhang

**Affiliations:** 1 Key Laboratory for Liquid-Solid Structural Evolution and Processing of Materials of Ministry of Education, Shandong University, Jinan 250061, People's Republic of China; 2 Key Laboratory of Special Functional Aggregated Materials, Ministry of Education, Shandong University, Jinan 250061, People's Republic of China; 3 Laiwu Yasai Ceramics Technology Co. Ltd. Laiwu 271100, People's Republic of China

**Keywords:** graphene platelets, B_4_C ceramic, pressureless-sintered, mechanical

## Abstract

B_4_C ceramics with different contents of graphene platelets (GPL) were synthesized by a pressureless process in Ar atmosphere. The influences of GPL on mechanical properties, thermal conductivity and electrical resistivity of the B_4_C ceramics were investigated. Mechanical properties ran up to optimal condition with hardness of 29.1 GPa, bending strength of 383.9 GPa and fracture toughness of 5.72 MPa m^1/2^ with 0.8 wt% GPL separately. Thermal conductivity and electrical resistivity reached extreme values of 26.35 W m^−1^ k^−1^ and 0.1 Ω cm^−1^. Performances of the ceramics were mainly affected by the generation of non-functional-GPL and the result indicated that a large amount of non-functional-GPL could contribute to poorer overall performance. Meanwhile, two particular pullout mechanisms concerning toughness enhancing was discussed in detail.

## 1. Introduction

B_4_C ceramics are widely used as nozzles, sharpeners and armours owing to their low density, high hardness, corrosion resistance and abrasion resistance. However, the relatively low fracture toughness and bending strength caused by the intrinsic brittleness of covalent bonding ceramics significantly reduce the service life of B_4_C ceramics [1–2]. Nowadays, B_4_C ceramics are mainly fabricated by hot pressing and spark plasma sintering (SPS) methods [3–7].

Graphene has extensive potential applications owing to its excellent properties in many aspects, such as outstanding mechanical properties (Young's modulus of 1100 GPa, bending strength of 125 GPa), thermal performance (super-high thermal conductivity of 5 × 10^3^ W m^−1^ K^−1^) and electrical properties (charge carrier mobility of 1.5 × 10^4^ cm^2^ V^−1^ s^−1^) [8–9]. However, it has been difficult to pursue the industrial-scale production of pure single-layered graphene up to now, taking its nature of agglomeration and cost-effect into consideration [10–12]. Fortunately, with the progress made in preparation technologies [13–15], graphene platelets (GPL) now can be used as reinforcement fillers in various bulk materials to improve their comprehensive properties, especially in ceramics [15–21]. Considerable research has been devoted to this filed. Earlier research carried out by Walker & Marotoo [16] found that fracture toughness of silicon nitride ceramics with the addition of 1.5 vol% GPL increased to 235% compared with the blank sample. Meanwhile, it was also observed that a new crack propagation path grew in the three-dimensional direction in the composites, which provided referencing basis for later research. Recently, Kovalčíková *et al*. [22] investigated the effects of GPL on the mechanical properties of B_4_C, where enhanced mechanical capability was achieved when 4.5 wt% GPL was added after hot pressing sintering. Yongqiang Tan *et al*. [23] prepared dense B_4_C/GPL composites heated in a vacuum using Ti_3_AlC_2_ as the additive and increased electrical conductivity of 250 S m^−1^ revealed with addition of 1 vol% GPL. In addition, the anisotropy of thermal conductivity of hot-pressed B_4_C/GPL composites was characterized by Pawel Rutkowski [24]. Crack deflection and pullout are the most typical toughening mechanisms in the last research, but B_4_C/GPL composites with excellent properties are laboratory-only at this stage. For pressureless sintered B_4_C ceramics, effects of GPL on the integrated performance of B_4_C, including mechanical properties, thermal properties and electrical behaviour are seldom studied.

In our work, B_4_C ceramics with different contents of GPL were synthesized by a liquid-state pressureless sintering method. The mixture powders of Al_2_O_3_/Dy_2_O_3_ and phenolic resin (PF) were used as sintering additives. Based on analysis of the mechanical, thermal and electrical tests, the most appropriate GPL content which contributes to dense B_4_C/GPL ceramics with superior properties has been determined.

## 2. Material and methods

Commercial pure powders of B_4_C (Mudanjiang Jingangzuan Boron Carbide Co. Ltd., D_50_ approx. 1.5 µm, purity greater than 97.0%) and GPL (Haifeng Vigon Materials Technology Co. Ltd., average layer thickness less than 3.0 nm, special surface area approx. 150 m^2^ g^−1^) were employed. PF (Laiwu Runda New Materials Co. Ltd.) was used as an organic adhesive. The Al_2_O_3_/Dy_2_O_3_ system, a maturing system that can increase the relative density of B_4_C ceramics [25], was used as the liquid-phase sintering aids. The composition of each sample is shown in table 1. Raw materials with a proper mass scale were homogenized in a ball-mill using Al_2_O_3_ as grind bodies in the anhydrous ethanol container for 24 h. After ball-milling, the well mixed and dried mixtures were ground, sieved and then shaped by die pressing process in a cylinder mould with a diameter of 65 mm and a height of 6 mm under 30 MPa. Next, the green bodies were pressed by an isostatic pressing machine under 200 MPa. The samples were heated at 2050°C for 1 h via pressureless sintering in Ar atmosphere with a heating rate of 10°C min^−1^.

**Table 1.** Ingredients and density of different samples.

	composition (wt%)						
sample	B_4_C	GPL	Al_2_O_3_ + Dy_2_O_3_	PF	theoretical density (g cm^–3^)	apparent density (g cm^–3^)	relative density (%)
1	100	0	4.00	15	2.56	2.47	96.48
2	100	0.4	4.00	15	2.56	2.46	96.09
3	100	0.8	4.00	15	2.56	2.46	96.09
4	100	1.0	4.00	15	2.55	2.45	96.08
5	100	1.2	4.00	15	2.55	2.43	95.29

Apparent density of the compact samples was measured based on the Archimedes principle. The electromechanical universal testing machine (CTN 5150, Shenzhen Suns Technology Co., Ltd.) was employed to calculate the mechanical properties of samples incised by inside diameter slicer at a loading speed of 0.5 mm min^−1^. The sizes of strip-sample used to obtain the fracture toughness and bending strength were 2 × 4 × 40 mm with an incision of 2 mm depth and 3 × 4 × 40 mm, respectively. Four items of each sample were measured to get a relativity precise value. Hardness was measured by a Vickers hardness instrument (DUV-1000, Shanghai Caikon Optical Instrument Co., Ltd) with a load of 4.90 N and loading time of 15 s. Four samples of the same composition were also tested. The feature of fracture micromorphology was characterized by a field emission high-resolution scanning electron microscope (SU-70, Hitachi). The thermal conductivity and electrical resistivity of polished samples were measured by hot-wire method through coefficient of thermal conductivity detector (TC–3000) and four-point probe resistance tester (KDY-1).

## 3. Results and discussion

### 3.1. The microstructure

**Figure 1.**
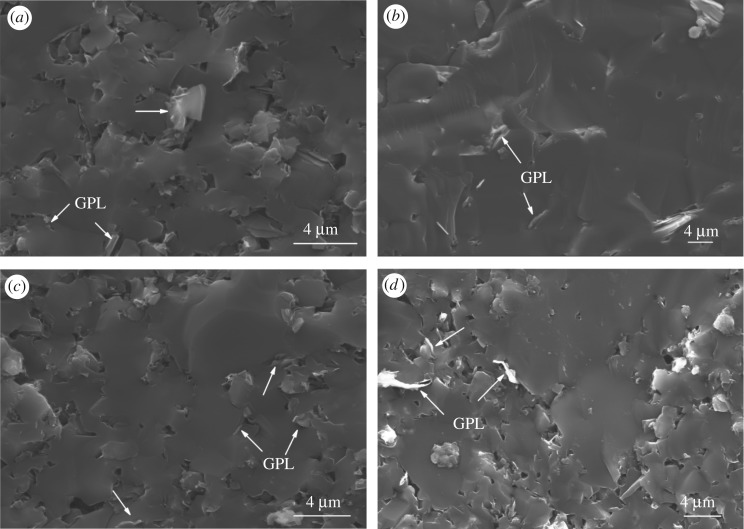
Microstructure of B_4_C samples with different amounts of GPL: (*a*) 0.4 wt%, (*b*) 0.8 wt%, (*c*) 1.0 wt%, (*d*) 1.2 wt%. The magnification of (*a*,*c*) and (*b*,*d*) is 4000, 3000, respectively.

**Figure 2.**
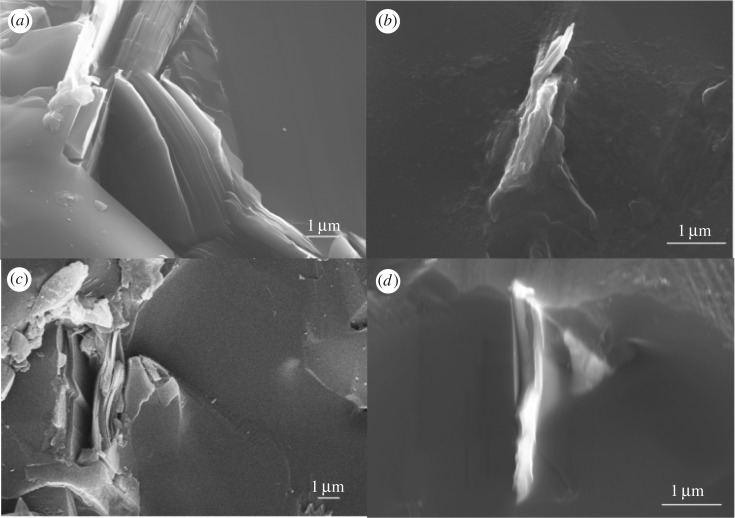
Detailed views of the B_4_C samples with different amounts of GPL: (*a*) 0.4 wt%, (*b*) 0.4 wt%, (*c*) 0.8 wt%, (*d*) 0.8 wt%. The magnification of (*a*,*c*) and (*b*,*d*) is 10 000, 20 000, respectively.

The apparent density and relative density of samples studied in this paper are listed in table 1, no obvious change can be observed. The microscopic images of the samples with different amounts of GPL from 0.4 wt% to 1.2 wt% are shown in figure 1. GPL are clearly marked in figure 1. Fractured surfaces of all samples with different GPL contents exhibit transgranular fracture, especially for the sample with 0.8 wt% GPL. With the content of GPL increasing, the porosity decreased first and then increased. Furthermore, it could be inferred that the B_4_C matrix and GPL are most closely linked at the level of 0.8 wt%. Some isolated GPL existing in the interstice of interstitial grain site begin to appear when 1.0 wt% GPL was added and this phenomenon gets worse when 1.2 wt% GPL was added. Closer combination between the B_4_C matrix and GPL could result in superior properties of composite ceramics, which means boost in performance could not be revealed obviously even if huge amounts of isolated GPL were added. In general, the isolated GPL could be regarded as the non-functional-GPL, the generation of which mainly results from the process of liquid-phase transfer. B_4_C ceramics usually could not be fully compacted after pressureless-sintering and pores would be left. During the process of liquid-phase mass transfer, GPL is always presented in the form of a lamellar solid as it is difficult for GPL to rearrange followed the liquid phase, leaving small amounts of tiny pores behind after liquid promoter additions cooled down (figure 2*a*). Therefore, the non-functional-GPL tends to appear along with pores (figure 1*d*). Few non-functional-GPL formed when its dosage increases from 0.4 wt% to 0.8 wt%, for this level of GPL can be evenly distributed in the B_4_C matrix. When the content rises to 1.0 wt%, it is harder for excessive GPL to be dispersed uniformly, so the GPL has a higher chance to combine with pores and thus non-functional-GPL is formed. In conclusion, the mass percentage of 0.8% is enough for GPL to be evenly distributed in the B_4_C ceramic basement and can be regarded as the optimal addition to promote densification of samples.

### 3.2. The mechanical properties of samples

**Figure 3.**
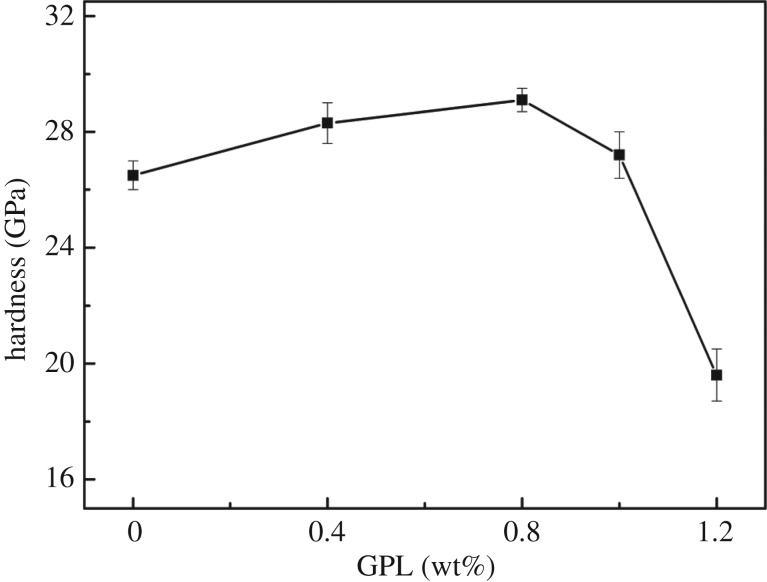
Influence of the GPL addition on hardness.

The relationship between different GPL contents and the hardness of samples are demonstrated in figure 3. The sample without GPL addition reached the hardness of 26.5 GPa. Moreover, the hardness increased immediately as the content of GPL rose to 0.8 wt%, peaking at the highest hardness of 29.1 GPa, and then showed a downward trend to its bottom at 19.6 GPa when the content of GPL is 1.2 wt%. The microstructural refinement shown in figure 1 may explain the microhardness variation. It can be assumed that aggregation of non-functional-GPL caused the final decline of hardness and appropriate GPL could integrate into the B_4_C matrix with few non-functional-GPL generated, which led to the increase in hardness.

**Figure 4.**
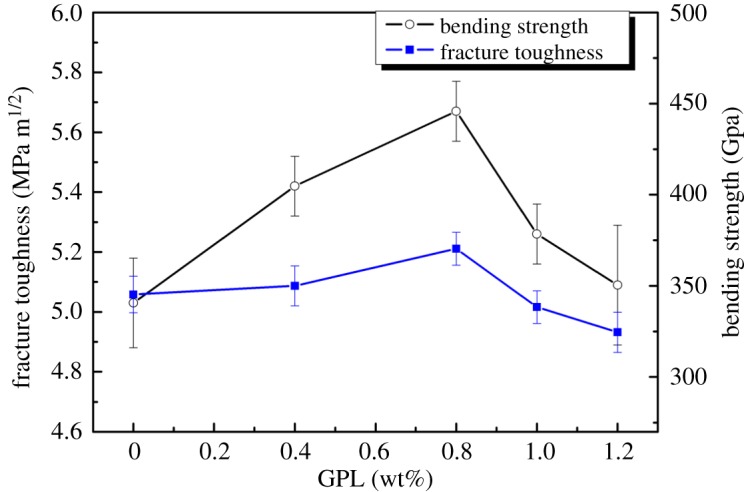
Influence of the GPL addition on fracture toughness and bending strength.

Variation trend of bending strength is actually the same as that of fracture toughness, displayed in figure 4. They all show an upward trend between 0 wt% and 0.8 wt%, during which time the value both peaked at 0.8 wt%, reaching 383.9 GPa and 5.72 MPa m^1/2^, respectively. After that, there was an obvious decline in both values from 0.8 wt% to 1.2 wt%, ending at 324.52 GPa and 5.09 MPa m^1/2^, respectively.

Analogous tendency of the apparent mechanical properties above has been reported in other investigations [21,22]. The enhanced toughness mainly results from the pullout of GPL shown in figure 2. As shown in electric mirror photos, the toughening mechanism of GPL is not completely equivalent to that of the pure single graphene or other fibres. There are two mechanisms when the pullout happens. Specifically, the whole GPL is pulled out when the fracture happens in one mechanism (figure 2*b*), showing a similar pullout mechanism of the carbon fibre [26]. In the other mechanism, the fracture process can be divided into two steps. Firstly, fracture occurs at the outermost graphene layers and then the pullout of GPL takes place between intimal slices of GPL (figure 2*c,d*), leaving a hole in the matrix. The latter mechanism should overcome the resistance from graphene layers and bonding force between graphene and the B_4_C substrate, which can absorb more rupture energy to prevent the continuing expanding of cracks.

### 3.3. The thermal and electrical properties of samples

**Figure 5.**
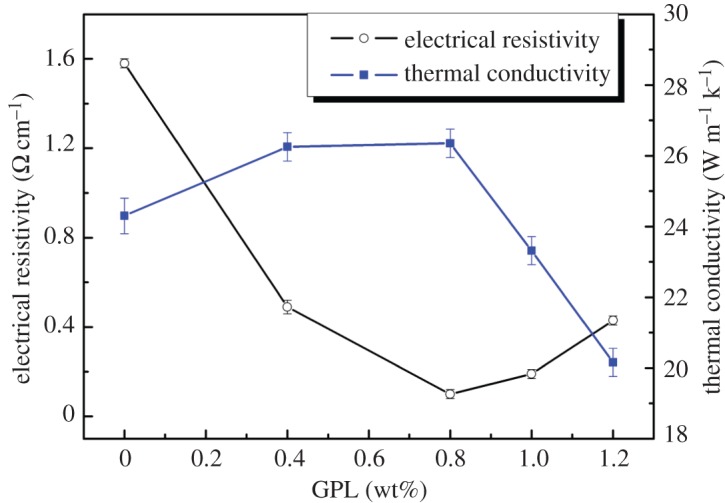
Influence of the GPL addition on thermal conductivity and electrical resistivity.

The effect of GPL addition on the room-temperature thermal conductivity and electrical resistivity is presented in figure 5. Composites with lower GPLs up to 0.8 wt% have the thermal conductivity in the range from 24.3 to 26.35 W m^−1^ k^−1^ and the even-distributed doped GPL should account for enhancement of the conductivity. For the specimen with 1.2 wt% GPL addition, the value of thermal conductivity decreased to 20.16 W m^−1^ k^−1^, which should be attributed to the increase of non-functional-GPL.

Minimum value of electrical resistivity was also obtained for a composite with 0.8 wt% of GPL, reaching 0.1 Ω cm^−1^. It was also verified that the value always showed a downtrend when the GPL content varied. This is because GPL is conducive to enhancing thermal conductivity and weakening the electrical resistivity, but the existence of the non-functional-GPL structure and increased pores have the opposite effect, when GPL larger than 0.8 wt%, increased non-functional-GPL structure and pores play a more important role. Furthermore, delivery of electron and vibrations in the lattice are constrained by these defects.

In general, for pressureless-sintered B_4_C/GPL ceramics, the density and mechanical capacity can catch up with the highest value of samples sintered by hot-pressing sintering or SPS; thermal conductivity and electrical resistivity also can be improved. Existence of non-functional-GPL is not conducive to taking advantage of the superb properties of GPL.

## 4. Conclusion

Compact B_4_C ceramics were obtained by a liquid-phase pressureless sintering method with different amounts of GPL. Effects of GPL on all-round properties including hardness, bending strength, fracture toughness, thermal conductivity and electrical resistivity were investigated. All properties studied have an improvement with addition of 0.8 wt% GPL with hardness of 26.5 GPa, bending strength of 383.9 GPa, fracture toughness of 5.72 MPa m^1/2^ and thermal conductivity of 26.35 W m^−1^ k^−1^. Lowest electrical resistivity of 0.1 Ω cm^−1^ is also achieved at this dosage of GPL. Further increase of these properties is hindered by the increasing non-functional-GPL. Two kinds of pullout mechanisms were discussed in detail.

## Date accessibility

The citing papers are available by clicking the hyperlink of the doi, and the raw data including original test data can be obtained by clicking the link: https://pan.baidu.com/s/1ggcazDL.
